# In‐Plane Anisotropy in van der Waals NiTeSe Ternary Alloy

**DOI:** 10.1002/advs.202410549

**Published:** 2025-01-13

**Authors:** Nguyen Huu Lam, Tae Gyu Rhee, Seongmun Kim, Byoung Ki Choi, Dang Nguyen Hoang, Ganbat Duvjir, Younghun Hwang, Jaekwang Lee, Young Jun Chang, Jungdae Kim

**Affiliations:** ^1^ Department of Physics University of Ulsan Ulsan 44610 Republic of Korea; ^2^ Department of Physics University of Seoul Seoul 02504 Republic of Korea; ^3^ Department of Smart Cities University of Seoul Seoul 02504 Republic of Korea; ^4^ Center for Spintronics Korea Institute of Science and Technology (KIST) Seoul 02792 Republic of Korea; ^5^ Department of Physics Pusan National University Busan 46241 Republic of Korea; ^6^ Advanced Light Source Lawrence Berkeley National Laboratory Berkeley CA 94720 USA; ^7^ Electricity and Electronics and Semiconductor Applications Ulsan College Ulsan 44610 Republic of Korea

**Keywords:** anisotropy, ARPES, DFT, NiTe_2_, NiTeSe, STM

## Abstract

The anisotropic properties of materials profoundly influence their electronic, magnetic, optical, and mechanical behaviors and are critical for a wide range of applications. In this study, the anisotropic characteristics of Ni‐based van der Waals materials, specifically NiTe_2_ and its alloy NiTeSe, utilizing a combination of comprehensive scanning tunneling microscopy (STM), angle‐resolved photoemission spectroscopy (ARPES), and density functional theory (DFT) calculations, are explored. Unlike 1T‐NiTe_2_, which exhibits trigonal in‐plane symmetry, the substitution of Te with Se in NiTe_2_ (resulting in the NiTeSe alloy) induces a pronounced in‐plane anisotropy. This anisotropy is clear in the STM topographs, which reveal a distinct linear order of charge distribution. Corroborating these observations, ARPES measurements and DFT calculations reveal an anisotropic Fermi surface centered at the $\bar{\Gamma }$ point, which is notably elongated along the k_y_ direction, leading to directional variations in in‐plane carrier velocities. Consequently, the Fermi velocity is highest along the k_x_ direction where the linear charge distribution aligns in real space and is lowest along the k_y_ direction. These findings offer valuable insights into the tunability of anisotropic properties in ternary transition metal dichalcogenide systems, highlighting their potential applications in the development of anisotropic electronic and optoelectronic devices.

## Graphical abstract

Unlike 1T‐NiTe_2_, which exhibits trigonal in‐plane symmetry, the substitution of Te with Se in NiTe_2_ (resulting in the NiTeSe alloy) induces a pronounced in‐plane anisotropy of linear charge order in scanning tunneling microscopy topographs. Angle‐resolved photoemission spectroscopy measurements reveal an anisotropic Fermi surface centered at the $\bar{\Gamma }$ point, leading to directional variations in in‐plane carrier velocities.

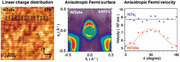

## 1 Introduction

Anisotropy, which manifests as directional variations in chemical and physical characteristics, plays a crucial role in a material's response to external stimuli, affecting a range of physical characteristics, including electrical, magnetic, optical, and mechanical behaviors.^[^ ^1^, ^2^, ^3^ ^]^ Layered van der Waals (vdW) materials form a unique class, distinguished by weak interactions between 2D atomic layers, often resulting in anisotropic behavior between in‐plane and out‐of‐plane directions. Conversely, within a 2D plane (in‐plane), vdW materials typically exhibit isotropic properties due to their symmetric crystal structures. For example, 2H‐MoSe_2_ displays in‐plane threefold rotational symmetry.^[^ ^4^, ^5^ ^]^ However, vdW materials with low in‐plane symmetry, such as ReS_2_ or ReSe_2_ (1T` or distorted 1T phase), exhibit distinct characteristics depending on the in‐plane crystal directions.^[^ ^6^, ^7^, ^8^, ^9^ ^]^ These anisotropic characteristics broaden the potential applications of these materials in electronic and optoelectronic devices. Novel phenomena, including the quantum spin Hall effect,^[^ ^10^, ^11^, ^12^ ^]^ valley polarization,^[^ ^13^, ^14^ ^]^ and ferromagnetism,^[^ ^1^, ^15^, ^16^, ^17^, ^18^ ^]^ have been discovered in such anisotropic materials. Therefore, methods are needed to induce or control anisotropy in these materials for advancing their technological applications.

Transition metal dichalcogenides (TMDs) exhibit a diverse range of physical properties, including superconductivity, topological insulator, valleytronics, spintronics, and catalytic activity.^[^ ^10^, ^19^, ^20^, ^21^, ^22^, ^23^, ^24^, ^25^, ^26^, ^27^, ^28^ ^]^ Recently, substitutional solid solutions forming ternary TMDs have emerged as a highly effective approach for tailoring various physical properties such as bandgap, band alignment, lattice parameters, and charge carrier type.^[^ ^29^, ^30^ ^]^ In ternary TMDs, one of the elements in the binary TMD structure is replaced by another element while maintaining a single homogeneous crystal phase. For instance, varying the chalcogen composition like MoS_2‐x_Se_x_ and WS_2‐x_Se_x_ allows for significant bandgap tuning across a broad spectral range.^[^ ^31^, ^32^, ^33^ ^]^ In some cases, alloying induces pronounced changes in crystal structure and electronic properties, such as a semiconductor‐to‐metal transition in WSe_2‐x_Te_x_ and WS_2‐x_Te_x_ or a Mott‐insulator‐to‐metal transition in 1T‐TaS_2‐x_Se_x_.^[^ ^34^, ^35^, ^36^, ^37^, ^38^ ^]^ Another example is WS_2‐x_Te_x_, which exhibits bipolar behavior, transitioning from the 2H phase (*p*‐type semiconductor) for *x* ≤ 1 to the 1T’ phase (*n*‐type semiconductor) for *x* ≥ 1.^[^ ^37^ ^]^ Given their tunable properties and potential for unprecedented material functionalities, research on ternary TMDs is expected to play an important role in the development of novel materials for advanced electronic, optoelectronic, and catalytic applications.

Recently, Ni‐based transition metal dichalcogenides (TMDs) such as NiTe_2_, NiSe_2_, and NiS_2_ have attracted attention due to their unique physical properties. Among them, bulk NiTe_2_ is particularly noteworthy as an ideal type‐II Dirac system, with its bulk Dirac point positioned near the Fermi level, distinguishing it from other members of this family.^[^ ^39^, ^40^, ^41^, ^42^, ^43^, ^44^ ^]^ A recent study reported that the strength of the spin‐orbit coupling (SOC) in NiTe_2_ can be tuned by Se substitution, enabling control over the energy level of the bulk Dirac point in NiTe_2‐x_Se_x_ ternary alloy.^[^ ^45^ ^]^ The SOC strength and the position of the bulk Dirac point exhibit nearly linear tunability, depending on the Se content in NiTe_2‐x_Se_x_. NiTe_2_ exhibits a 1T‐layered structure, whereas NiSe_2_ and NiS_2_ adopt a pyrite structure. Such structural disparity presents interesting possibilities for exploring anisotropy that could emerge in ternary TMD alloys combing these distinct phases.

In this study, we investigate the anisotropic properties of NiTe_2_ and NiTeSe single crystals, characterized using scanning tunneling microscopy (STM), angle resolved photoemission spectroscopy (ARPES), and density functional theory (DFT) calculations. STM topography shows that NiTe_2_ maintains a typical trigonal in‐plane lattice structure consistent with its high symmetry. In contrast, NiTeSe ternary alloy exhibits pronounced anisotropic linear features, disrupting the inherent threefold symmetry. Both ARPES measurements and DFT calculations consistently show an elliptical Fermi surface near the $\bar{\Gamma }$ point in NiTeSe, resulting in anisotropic Fermi velocities that vary with the direction of in‐plane k vectors. The substitution of Se in NiTe_2_ modifies the crystal symmetry and enhances anisotropic properties, suggesting potential applications in electronic and optoelectronic devices.

## 2 Results and Discussion

**Figure 1:**
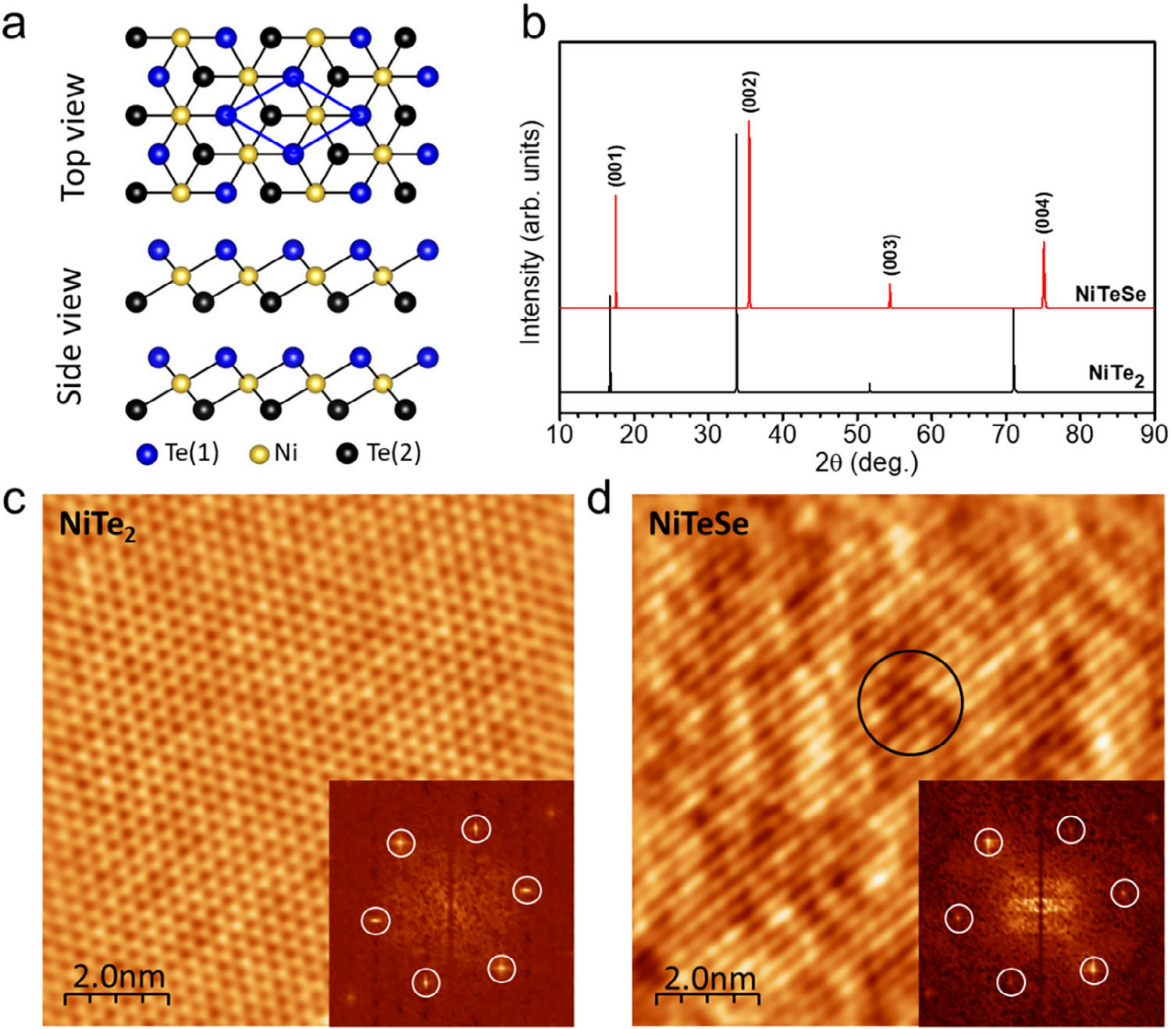
NiTe_2_ and NiTeSe single crystals. a) Structural model of 1T‐NiTe_2_. b) XRD data of the cleavage plane of NiTe_2_ and NiTeSe single crystals. Atomically resolved STM topography and corresponding FFT (inset) images of c) NiTe_2_ and d) NiTeSe. White circles in the FFT insets indicate the hexagonal lattice. Scanning conditions: c) *V*_b_ = 1.0 V, *I*_t_ = 30 pA and d) *V*_b_ = 0.6 V, *I*_t_ = 30 pA.

Bulk NiTe_2_ crystallizes in the CdI_2_‐type trigonal (1T) structure with a P $\bar{3}$ m1 space group (**Figure** **1**a).^[^ ^46^ ^]^ In this layered structure, Te‐Ni‐Te monolayers are alternately stacked by weak van der Waals forces, while hexagonally close‐packed Ni atoms are octahedrally coordinated by six Te atoms within each Te‐Ni‐Te slab. NiTe_2_ and NiTeSe single crystals are synthesized, and their structures are confirmed using X‐ray diffraction (XRD) and STM. Figure 1b presents the XRD results for NiTe_2_ and NiTeSe crystals, showing well‐oriented growth along the *c*‐axis and confirming the high crystallinity of the 1T phase. Furthermore, STM topographs and corresponding fast Fourier transformation (FFT) images (insets) of NiTe_2_ (Figure 1c) and NiTeSe (Figure 1d) show hexagonal lattice structures (hexagonal lattice peaks are marked by white circles in the FFT insets). Substituted Se atoms in NiTeSe alloy appear as dark regions in Figure 1d (marked by a circle), attributed to the smaller atomic size of Se compared to Te. Interestingly, a distinctive linear feature along the diagonal direction was observed on the NiTeSe surface (Figure 1d), which will be discussed later.

**Figure 2:**
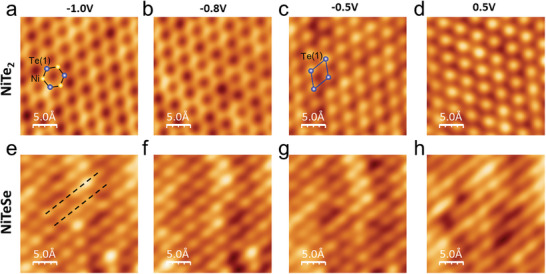
Bias‐dependent STM images of NiTe_2_ and NiTeSe surfaces. STM topographs of a–d) NiTe_2_ and e–h) NiTeSe at different bias voltages. Linear features are marked by black dashed lines. All images: *I*_t_ = 30 pA.

Bias‐dependent STM images of NiTe_2_ and NiTeSe surfaces are shown in **Figure** **2**. Notably, for 1T‐NiTe_2_, a transition from a honeycomb (Figure 2a,b) to triangular structures (Figure 2c,d) is observed at a bias voltage below −0.5 V (see Figure S1, Supporting Information). In STM topography of TMDs, the top chalcogen layer typically dominates the atomistic features, resulting in a triangular lattice structure. However, the bias‐switched topography (Figure S2, Supporting Information) and DFT‐simulated STM images (Figure S3b,c, Supporting Information) suggest that a honeycomb pattern arises from the contribution of Ni *d*‐electron states below −0.5 V, agreeing well with the observed bias‐dependent STM images.

In contrast to the NiTe_2_, the alloyed NiTeSe presents a prominent linear feature (marked by black dashed lines) across all bias voltages, though the intensity of the linear feature varies with applied bias voltage (Figure 2e–h; Figure S4, Supporting Information). Comparing STM topography with simultaneously obtained d*I*/d*V* mapping images of NiTeSe (Figure S5, Supporting Information), it is evident that these linear features become more pronounced in the d*I*/d*V* mapping images, suggesting the presence of anisotropic charge distribution along the diagonal direction. To understand the observed anisotropy in NiTeSe, it is essential to investigate the energy band structure of the system.

**Figure 3:**
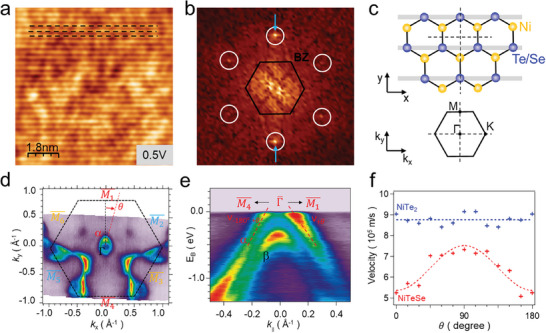
Anisotropy in NiTeSe. a) STM topography and b) corresponding FFT images of NiTeSe. White circles in the FFT image indicate the hexagonal lattice. The first Brillouin zone (BZ) is added (black hexagon). c) Structure model of top Te/Se and middle Ni atoms in NiTeSe and its relationship to hexagonal BZ. Linear features are marked by gray shaded lines. d) Fermi energy contour of NiTeSe derived from ARPES. e) Energy dispersion curves of NiTeSe follow the path $\bar{M_{1}}-\bar{\Gamma }-\bar{M_{4}}$ Two bands are observed, indicated as *α* and *β*. f) Absolute value of velocities, averaged along each direction $\left({\bar{V}}_{\theta }=\frac{\left|V\;+\;\theta |+\right|V\;-\;180^{\circ }\;+\;\theta |}{2}\right)$, for an *α* band at *E*_B_ = 0 eV from *θ* = 0° to 180°.

**Figure** **3**a,b presents topography and corresponding FFT images of NiTeSe, respectively. The intensity of two lattice peaks in the FFT image (marked by blue arrows in Figure 3b) was substantially enhanced, reflecting the anisotropy in the topography. The structural model of NiTeSe (showing the top Te/Se and middle Ni atoms) and corresponding hexagonal Brillouin zone (BZ) are provided in Figure 3c. The linear feature (shaded gray line) aligns with one of the Γ‐K directions (or the k_x_ direction), breaking threefold rotational symmetry, as shown in Figure 3a,c. This low symmetry suggests that the NiTeSe system may exhibit anisotropic characteristics of charge carriers between the k_x_ and k_y_ directions.

We conducted ARPES measurements to investigate the energy dispersion in momentum space. Figure 3d,e displays a Fermi surface map and the energy dispersion curve along the $\bar{M_{1}}-\bar{\Gamma }-\bar{M_{4}}$ direction of NiTeSe, respectively. The results reveal two hole‐like bands, referred to as *α* and *β* bands in Figure 3e, centered at the $\bar{\Gamma }$ point, and the *α* band crosses the Fermi energy as shown in Figure 3d,e. Intriguingly, in Figure 3d, the *α* band presents an elliptical energy contour oriented with its long axis along the k_y_ axis and its short axis along the k_x_ axis. This anisotropic dispersion of the *α* band leads to variations in carrier velocities. Figure 3f shows the Fermi velocities ($\bar{v_{\theta }}$) extracted from the slope of the *α* bands of NiTe_2_ and NiTeSe as a function of angle *θ* (as defined in Figure 3d) with respect to $\bar{M_{1}}$. The Fermi velocity is maximized at *θ* = 90° (along the Γ‐K direction or k_x_ direction), which corresponds to the direction of linear feature observed in STM topography (Figure 3a–c). A detailed analysis of velocity extraction from the energy dispersion curves for NiTeSe is provided in Figure S6 (Supporting Information).

**Figure 4:**
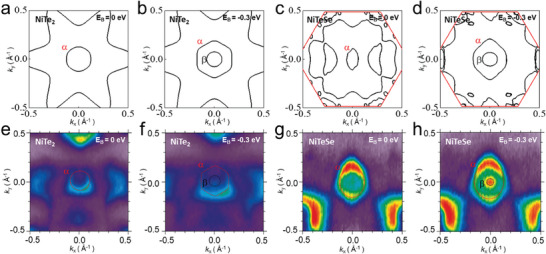
Comparison between DFT calculations and ARPES results. a–d) Calculated Fermi surface and e–h) constant energy contour maps of NiTe_2_ and NiTeSe at *E*_B_ = 0 and −0.3 eV.

Experimental STM and ARPES observations are further corroborated by DFT calculations. **Figure** **4**a–h presents DFT calculation and ARPES results for constant energy contours at various binding energies (*E*_B_), respectively. For the *α* band centered at the $\bar{\Gamma }$ point, NiTe_2_ exhibits an isotropic circular constant energy contour at the Fermi energy (*E*_B_ = 0 eV, Figure 4a,e), which evolves into a hexagonal shape at *E*_B_ = −0.3 eV (Figure 4b,f) as expected in the trigonal crystal symmetry of NiTe_2_. This isotropic behavior accounts for the uniform Fermi velocities as a function of *θ* as shown in Figure 3f. In contrast, in both Figure 4c,g,d,h, the constant energy contours of NiTeSe display a strongly elongated ellipse along the k_y_ direction for the *α* band, indicating broken threefold symmetry. Given that the Fermi velocity is proportional to the slope of *dE/dk*, it is expected to be slowest along the k_y_ direction (at *θ* = 0° in Figure 3f) and fastest along the k_x_ direction, where the slope is steepest (at *θ* = 90° in Figure 3f). These anisotropic characteristics are consistently confirmed in both experimental and theoretical DFT calculations for NiTeSe, as shown in Figures 4 and 3f.

The atomic radius of Se is ≈100 pm, while that of Te is ≈120 pm, a substantial 20% size difference. This atomic size disparity disrupts the threefold symmetry of NiTe_2_ when Se is incorporated, leading to a distinctive in‐plane anisotropic charge distribution and band structure in NiTeSe alloy. Furthermore, the simulated STM image suggests that the orbital ordering between Te and Se atoms might play an important role in the observed anisotropy (Figure S9, Supporting Information). Our findings suggest that the in‐plane anisotropy in vdW materials can be effectively controlled via substitutional alloying (Figures S8 and S10, Supporting Information). Tunable anisotropic electronic properties are anticipated to have a significant impact on various applications, enabling the selective increase or decrease of electrical or thermal conductivity in specific directions. This capability is particularly important for advancements in spintronics, superconductivity, nanoscale metal interconnectors, thermoelectricity, and optoelectronic devices.

## 3 Conclusion

Our comprehensive investigation demonstrates that substituting Se in NiTe_2_ significantly alters its electronic structure and enhances anisotropic properties. The introduction of Se disrupts the inherent threefold rotational symmetry, leading to a distinctive linear feature in STM topography, indicative of an anisotropic charge distribution. Accordingly, an energy band centered at the $\bar{\Gamma }$ point is strongly elongated along the k_y_ direction in NiTeSe, as confirmed by both ARPES measurements and DFT calculations. This anisotropic band dispersion results in notable variations in carrier velocities, with the slowest occurring along the k_y_ direction and the fastest along the k_x_ direction. The anisotropic property achieved through Se substitution enhances the potential of NiTe_2‐x_Se_x_ ternary alloys for advanced electronic and optoelectronic applications, where directionally dependent electronic behaviors are crucial. This study highlights the critical role of compositional modifications in tailoring the physical properties of layered transition metal dichalcogenides.

## 4 Experimental Section

### Sample Preparation

Single crystals were grown by using the vertical Bridgman gradient solidification method. For the preparation of NiTe_2‐x_Se_x_ single crystals, high‐purity nickel (Ni‐99.99%) powder, tellurium (Te‐99.99999%), and selenium (Se‐99.9999%) shots as starting materials were used. The elements were placed into an ampoule with a diameter of ≈10 mm and a length of 15 mm, which featured a capillary bottom to promote the growth of a single seed. Before loading, the inner wall of the quartz ampoule was coated with carbon. The ampoule was then evacuated to <10^−6^ Torr and sealed. After encapsulation, the sealed ampoule was mixed and loaded into a vertical furnace. Initially, the furnace temperature was raised to 600 °C and held there for 72 h to mitigate excessive pressure buildup due to the high vapor pressures of Se, which could otherwise cause an explosion during NiTe_2‐x_Se_x_ formation. Subsequently, the temperature was increased to 1150 °C at a rate of 10 °C h^−1^, then held for 7 days. (Note: The melting point of Ni is 1455 °C.) For single‐crystal growth, the temperature was gradually reduced to room temperature over a period of 10 days. The resulting single crystals measured ≈5 × 5 × 1 mm^3^. The mole fraction, *x*, was determined by energy‐dispersive X‐ray spectroscopy (EDS) using field‐emission scanning electron microscopy (FE‐SEM 6500, JEOL) (Figure S11, Supporting Information).

### Scanning Tunneling Microscopy and Spectroscopy Measurements

Scanning tunneling microscopy (STM) measurements were performed using a low‐temperature, homemade STM at 79 K under a base pressure of ≈7 × 10^−11^ Torr.^[^ ^47^ ^]^ Tungsten tips were electrochemically etched and cleaned in situ by electron beam heating. NiTe_2_ and NiTeSe single crystals were cleaved in situ to obtain clean surfaces for STM measurements. The constant‐current mode with the bias voltage applied to the sample was used for collecting all STM topographies. Differential conductance (d*I*/d*V*) mapping images were measured using a standard lock‐in technique with a modulation signal of 10 mV at a frequency of 1.2 kHz.

### Angle‐Resolved Photoemission Spectroscopy Measurements

All angle‐resolved photoemission spectroscopy (ARPES) measurements were conducted at the micro‐ARPES end‐station, with a base pressure of ≈4 × 10^−^¹¹ Torr, located at the MERLIN facility on beamline 4.0.3 of the Advanced Light Source at Lawrence Berkeley National Laboratory. The ARPES setup featured a Scienta R4000 electron analyzer. Single crystals of NiTe_2‐x_Se_x_ were mounted on ARPES sample holders and covered using silver epoxy and ceramic top posts. These samples were then transferred and cleaved in situ at temperatures below 20 K to ensure pristine surfaces. Throughout the ARPES measurements, the sample temperature below 20 K and energy and angle resolutions higher than 20 meV and 0.1°, respectively, were set. Measurements were performed using a range of photon energies between 80 and 150 eV.

### Theoretical Calculations

All first‐principles calculations were performed based on DFT with the projector augmented wave (PAW) method,^[^ ^48^ ^]^ as implemented in the Vienna Ab‐initio Simulation Package (VASP). The Perdew–Burke–Ernzerhof (PBE) functional within the Generalized Gradient Approximation (GGA)^[^ ^49^ ^]^ was employed for exchange‐correlation, and the optB86b‐vdW functional was used to account for van der Waals interactions. The cut‐off energy for the self‐consistent field (SCF) loop was set to 10^−8^ eV, and the structures were fully relaxed until the forces were less than 10^−8^ eV Å^−1^. A 1 × 1 × 1 unit cell $(P\bar{3}m1$) was used for NiTe_2_, and a 2 × 2 × 1 supercell was selected for NiTeSe in the electronic calculations. The reciprocal space of each structure was sampled using a Γ‐centered k‐mesh, with 8 × 8 × 6 for NiTe_2_ and 4 × 4 × 6 for NiTeSe. The electronic properties of both materials were calculated with spin‐orbit coupling (SOC), and the Fermi surface was analyzed using the FermiSurfer.^[^ ^50^ ^]^

## Conflict of Interest

The authors declare no conflict of interest.

## Supporting information



Supporting Information

## Data Availability

The data that support the findings of this study are available from the corresponding author upon reasonable request.
