# Supplementary material for: In‐Plane Anisotropy in van der Waals NiTeSe Ternary Alloy
Source: Adv Sci (Weinh). 2025 Jan 13;12(9):2410549. doi: 10.1002/advs.202410549 (PMC11884612; doi:10.1002/advs.202410549)
Supplement: Supplementary file 1 — Supporting Information [file ADVS-12-2410549-s001.docx]

**Supporting Information**

**In-plane anisotropy in van der Waals NiTeSe ternary alloy**

*Nguyen Huu Lam, Tae Gyu Rhee, Seongmun Kim, Byoung Ki Choi, Dang Nguyen Hoang, Ganbat Duvjir, Younghun Hwang^*^, Jaekwang Lee^*^, Young Jun Chang^*^, Jungdae Kim^*^*

N. H. Lam, D. N. Hoang, G. Duvjir, J. Kim

Department of Physics, University of Ulsan, Ulsan 44610, Republic of Korea

Email: kimjd@ulsan.ac.kr

T. G. Rhee, B. K. Choi, Y. J. Chang

Department of Physics, University of Seoul, Seoul 02504, Republic of Korea

Email: yjchang@uos.ac.kr

T. G. Rhee, Y. J. Chang

Department of Smart Cities, University of Seoul, Seoul 02504, Republic of Korea

T. G. Rhee

Center for Spintronics, Korea Institute of Science and Technology (KIST), Seoul 02792, Republic of Korea

S. Kim, J. Lee

Department of Physics, Pusan National University, Busan 46241, Republic of Korea

Email: jaekwangl@pusan.ac.kr

B. K. Choi

Advanced Light Source, Lawrence Berkeley National Laboratory, Berkeley, California 94720, United States

Y. Hwang

Electricity and Electronics and Semiconductor Applications, Ulsan College, Ulsan 44610, Republic of Korea

Email: younghh@uc.ac.kr

N. H. Lam, T. G. Rhee, and S. Kim contributed equally to this work.

Keywords: NiTeSe, NiTe_2_, Anisotropy, STM, ARPES, DFT

In the bias dependence, **Figure S1**, two distinct surface structures in NiTe_2_ can be observed. The honeycomb structure can be resolved for filled states with bias voltages below -0.5 V, as shown in Figure S1a-c. At bias voltages range of V ≥ -0.5 V, the typical triangular structure is observed (Figure S1d-j).


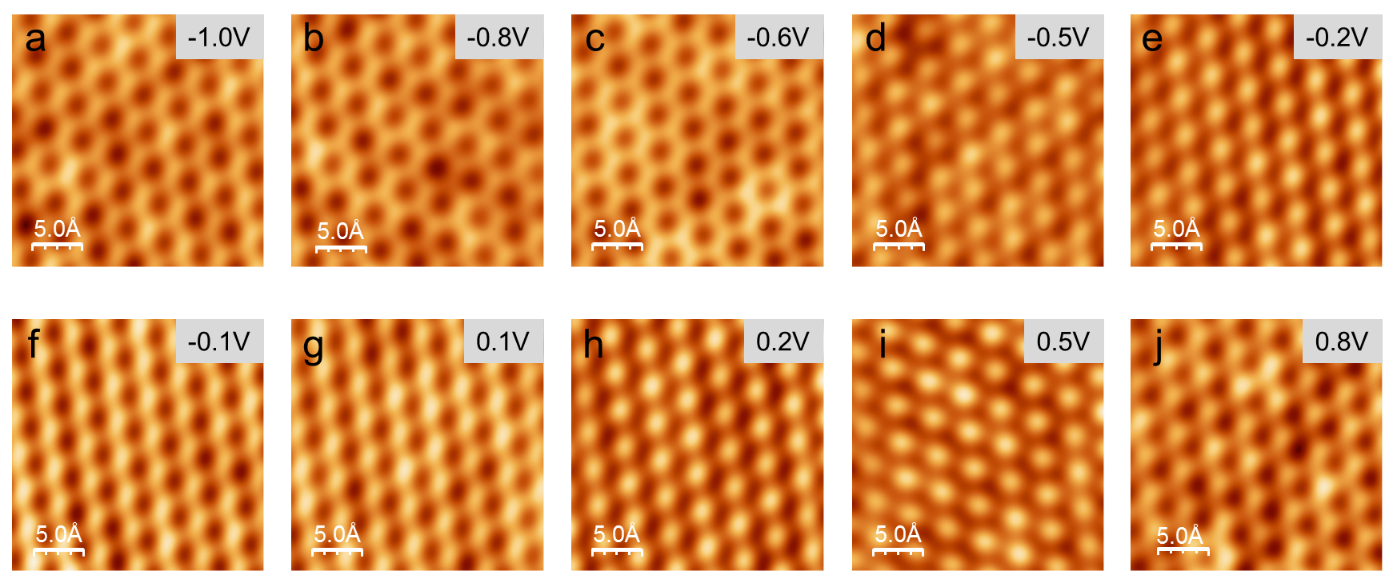


**Figure S1**. Bias dependent images of NiTe_2_ from the empty state (positive sample bias) to filled state (negative sample bias). All images: *I*_t_ = 30 pA.

Bias-dependent STM images of NiTe_2_ reveal that triangular or honeycomb structures can be selectively imaged by adjusting the sample bias. **Figure S2** shows a bias-switched image where the bias voltage is changed from -0.1 V to -0.6 V, resulting in a transition from a triangular to a honeycomb structure. The bright spots correspond to the topmost Te (Te(1)) atoms. Within the primitive unit cell (blue rhombus) formed by four Te(1) atoms, two hollow sites with varying contrast are identified, likely corresponding to Ni and bottom Te atoms based on the 1T-NiTe₂ model (Figure 1a). The atomic structure over the STM image at the bottom of Fig. S2 indicates that the honeycomb structure is formed by Te(1) and Ni atoms.


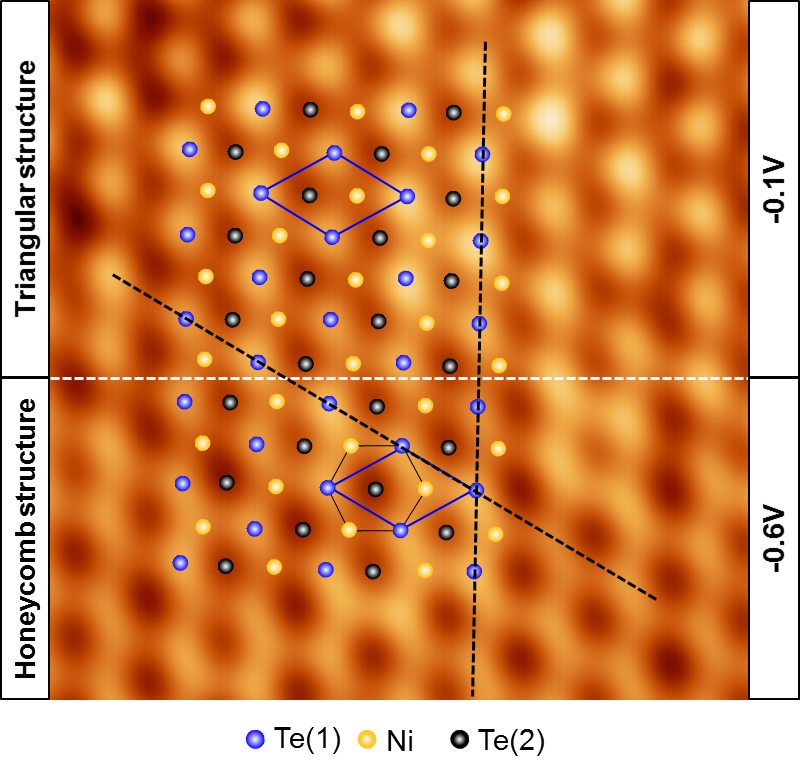


**Figure S2**. Bias-switched image of NiTe_2_. The bias voltage is switched from -0.1 V to -0.6 V while scanning with a 30 pA current. Size of image: 5 x 5 nm^2^. The atomic model of NiTe_2_ is superimposed where blue, black, and yellow balls represent the top Te (Te(1)), bottom Te (Te(2)), and Ni atoms, respectively. The blue rhombuses represent primitive unit cells.

STM topography and DFT simulation images of NiTe_2_ are presented in **Figure S3**a and S3b, respectively, showing either triangular (upper panel at -0.1 V) or honeycomb (lower panel at -0.6 V) lattices. The results indicate that the triangular lattice is attributable to the top Te (Te(1), blue balls), whereas the honeycomb structure arises from both triangular Te(1) and Ni (yellow balls) lattices. This is further supported by the charge density distribution plots in Figure S3c. In the bias range above -0.5 V, charge predominantly accumulates on Te atom sites, favoring the emergence of the triangular Te(1) lattice in the STM topography. At bias values below -0.5 V, there is an additional contribution from Ni *d*-electron states, resulting in the formation of the honeycomb structure.


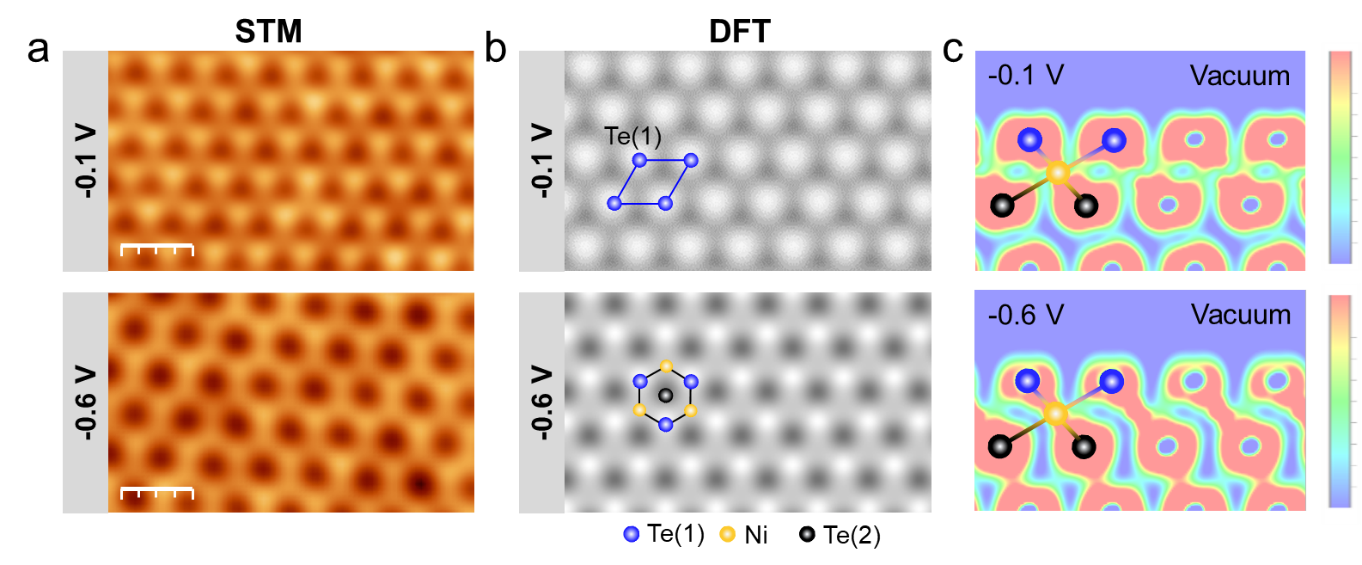


**Figure S3**. a) STM images, b) DFT simulated images and c) charge-density plots of triangular (upper panels) and honeycomb (lower panels) structures of NiTe_2_. Scale bars are 5.9 Å.

Bias-dependent STM images of NiTeSe surfaces are presented in **Figure S4**. The linear feature (marked by black dashed lines) is observed for all bias voltages.


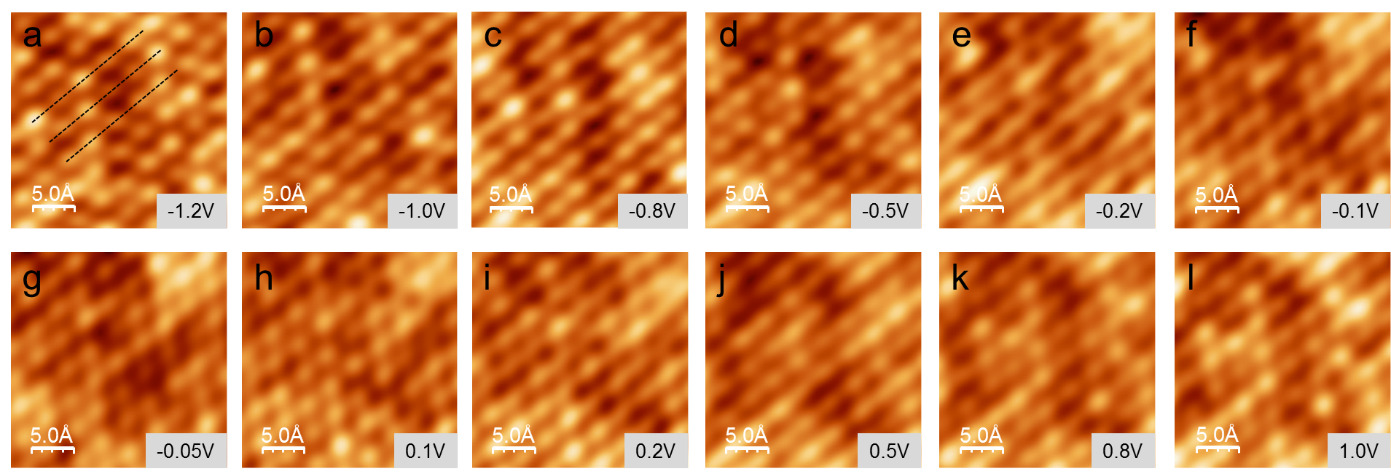


**Figure S4**. Bias dependent images of NiTeSe. Linear features are marked by black dashed lines. All images: *I*_t_ = 30 pA.

To clarify the origin of the linear features on the NiTeSe surface, we conducted d*I*/d*V* mapping images. **Figure S5** displays STM topography and simultaneous d*I*/d*V* mapping images of NiTeSe surface at different bias voltages, both showing identical linear features (marked by black dashed lines). The d*I*/d*V* mapping images highlight that these linear features become more pronounced, suggesting an anisotropic charge distribution along the diagonal direction.


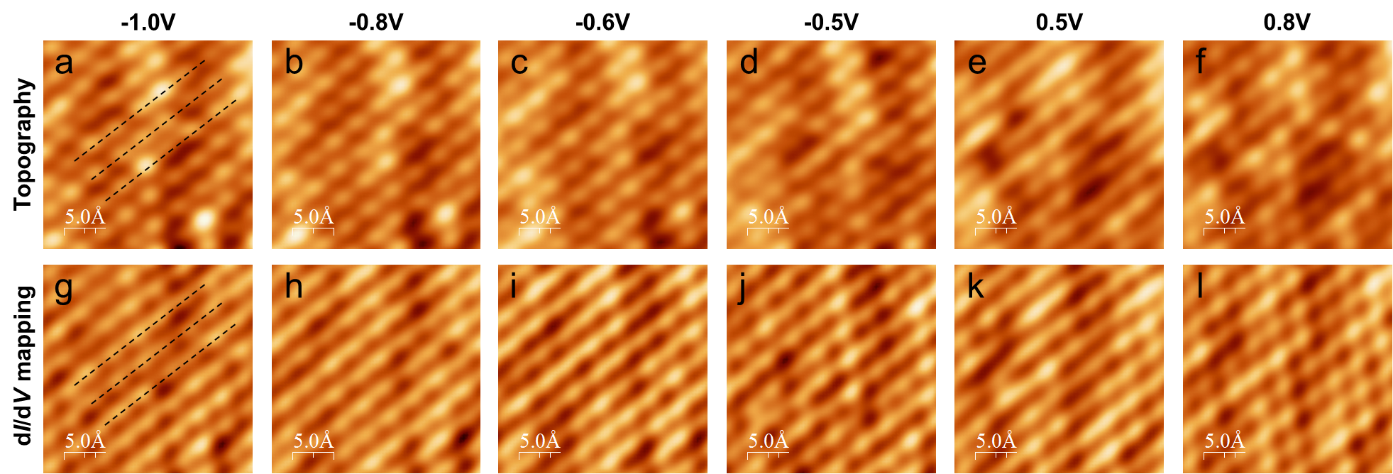


**Figure S5**. a-f) STM topography and g-l) simultaneously taken d*I*/d*V* mapping images of NiTeSe surface at different bias voltages. Linear features are marked by black dashed lines. All images: *I*_t_ = 30 pA.


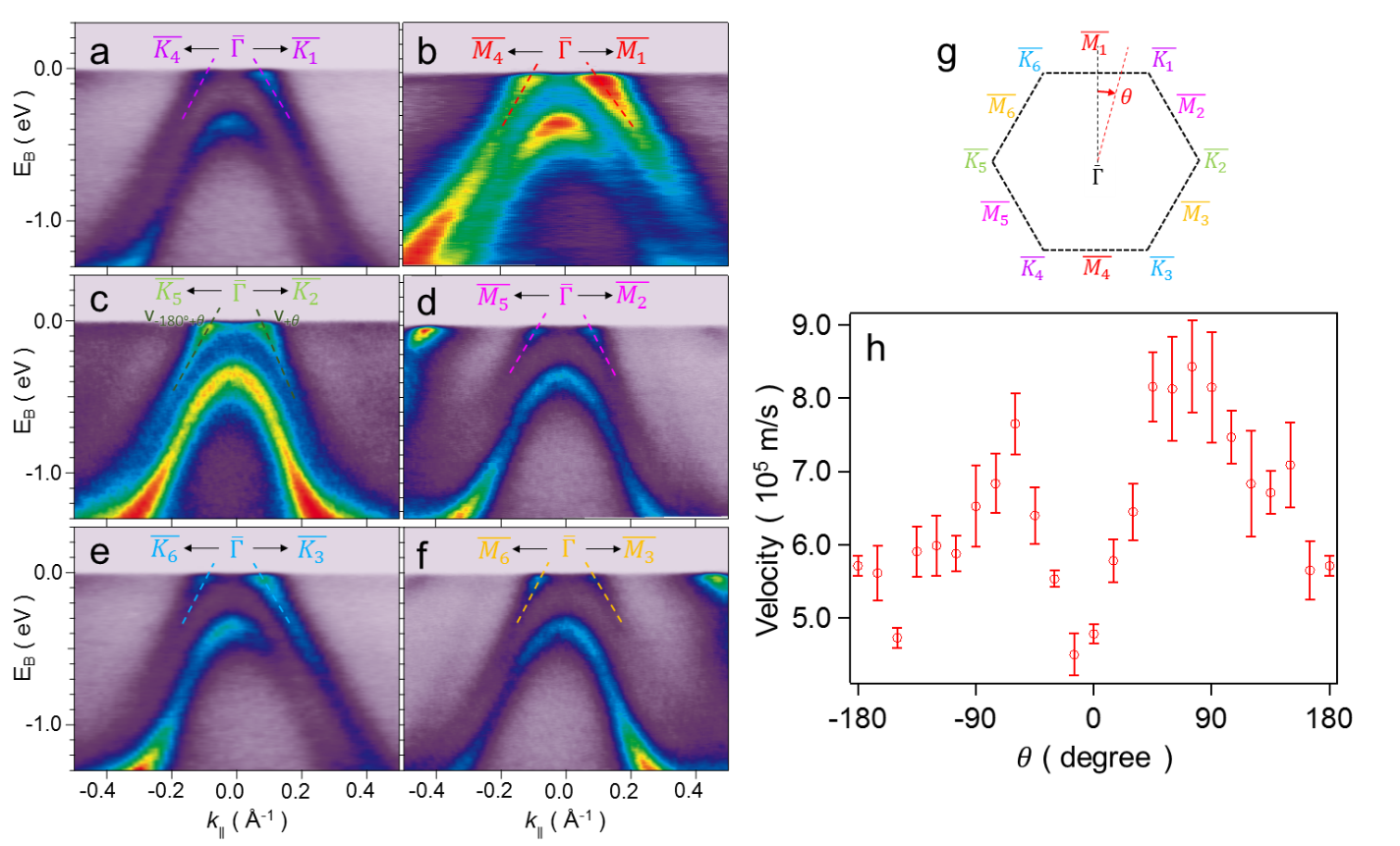


**Figure S6**. a-f) Energy dispersion curves of NiTeSe along all high symmetric directions. Velocity was extracted by linear fitting at some point (*E*_B_, *k*) near the Fermi level. g) Hexagonal Brillouin zone. h) Absolute value of velocities, averaged along each direction ($\bar{\text{v}_{\theta}}$ = $\frac{\text{|v}\text{+}\text{θ}\text{| + |v}\text{-180°+}\text{θ}\text{|}}{2}$), (-180°≤ 𝜃 ≤ 180°).

The energy band structures of NiTe_2_ and NiTeSe alloy are shown in **Figure S7**. For NiTe_2_, the α and *β* bands near *E*_B_ = -0.3 eV exhibit isotropic evolution along the *M* and *K* directions. In contrast, NiTeSe shows a relatively elongated evaluation of the α band along the *M* direction, while the *β* band maintains isotropic behavior. These anisotropic characteristics are consistent with the calculated Fermi surface and ARPES results of NiTe_2_ and NiTeSe.


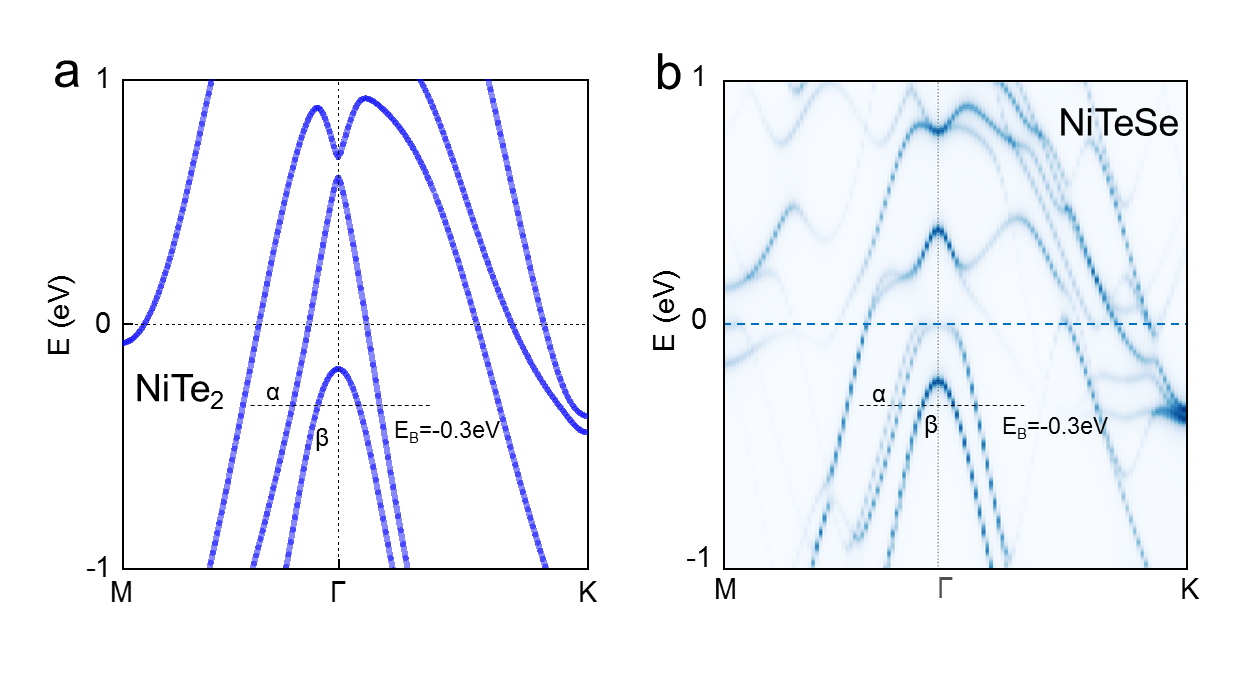


**Figure S7**. The simulated energy band structures of a) NiTe_2_ and b) NiTeSe. The Fermi level is set to zero.

**Figures S8**a-c show the Fermi surface contours of NiTe_2_, NiTe_1.75_Se_0.25_, and NiTeSe, respectively. Each contour displays the Γ-centered α band with differing degrees of anisotropy. To quantify the anisotropy of the contours, we calculated the b/a ratio in Figure S8d, where a and b represent the short and long radii of the contour, respectively. As the Se substitution increases, the ratio also increases, reflecting enhanced anisotropy. Furthermore, we analyzed the absolute values of the Fermi velocities along different directions in Figure S8e. These results indicate that NiTe_1.75_Se_0.25_ exhibits anisotropic contour and velocity with a lesser extent than NiTeSe, and the amount of Se influences the in-plane anisotropy.


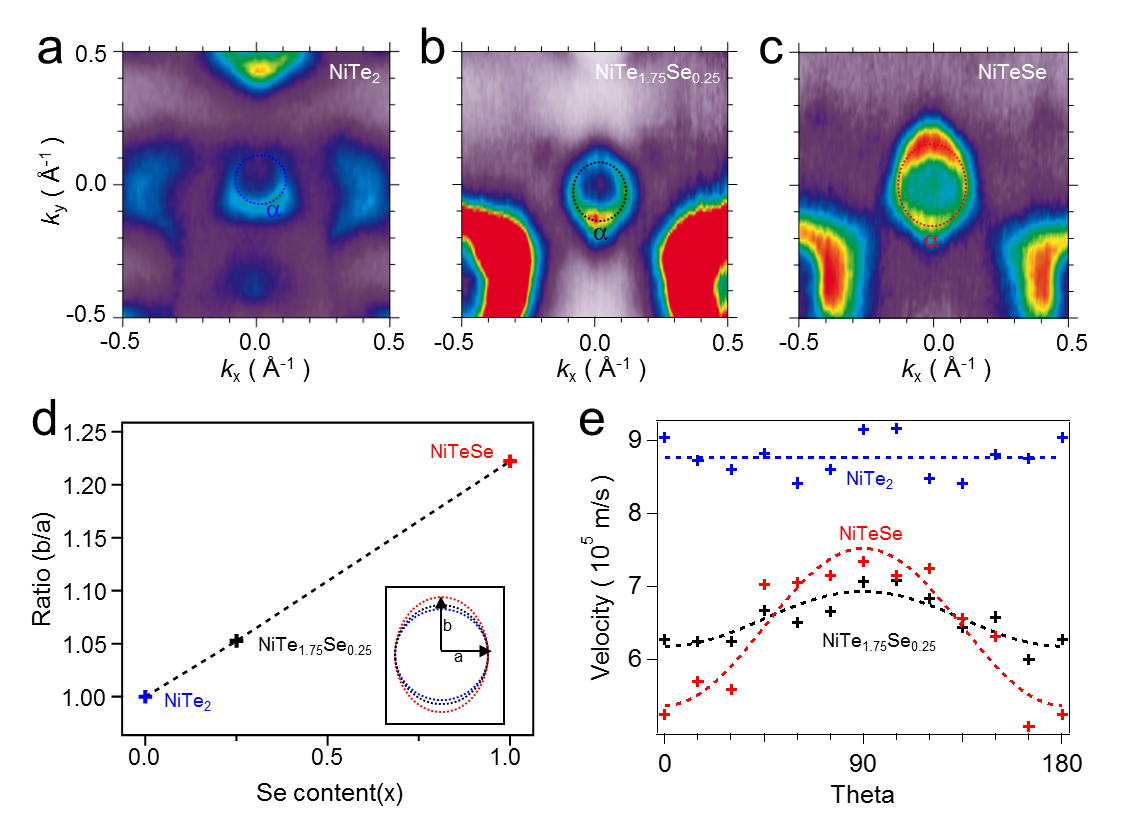


**Figure S8.** **Se** s**ubstitution-dependent anisotropy of electronic structure of NiTe_2-x_Se_x_ (*x* = 0, 0.25, 1).** a-c) Fermi surface contour of α band for each substitution level. d) Anisotropic ratio derived from each contour in (a-c). e) Absolute value of Fermi velocities along different directions for each sample.

**Figure S9**a presents the STM image simulation results^[1,2]^ for NiTeSe, revealing a linear ordering of charge distribution along the a-axis, where Te and Se atoms are alternately connected, rather than the b-axis, where Te and Se atoms are separately aligned. The simulation findings suggest that the orbital ordering between Te and Se atoms might play an important role in the observed anisotropy.


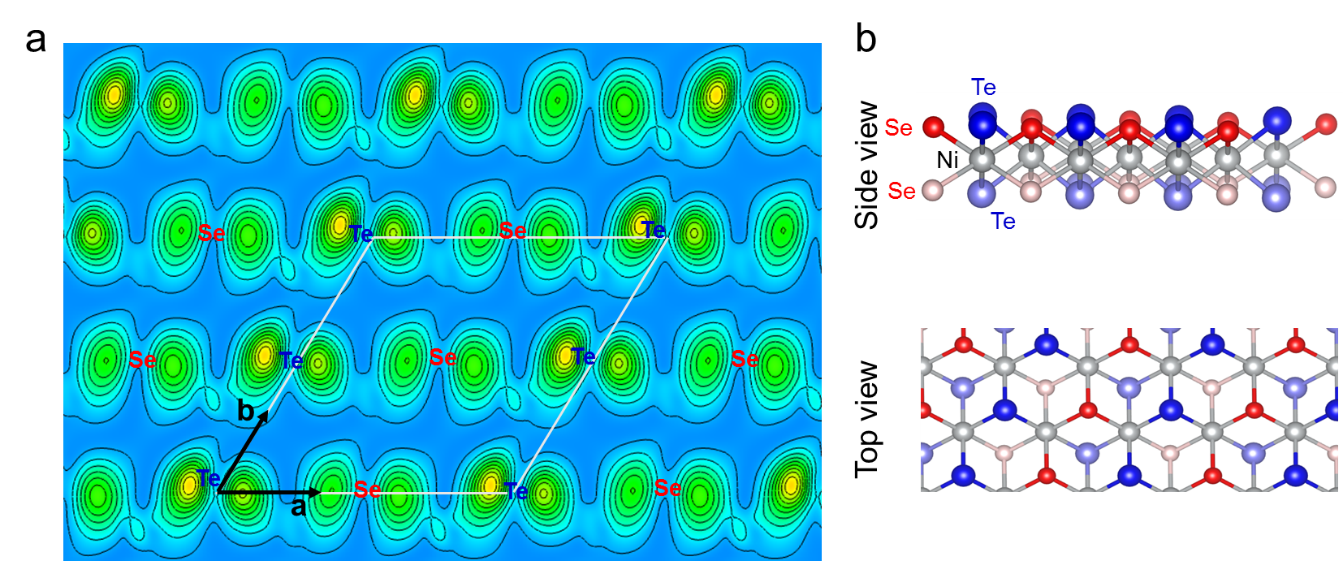


**Figure S9**. a) Simulated STM image of NiTeSe and b) structure model of NiTeSe.

**Figure S10** presents DFT calculation for the Fermi surface of NiTe_2_, NiTeSe, and NiTeS. The atomic radius of Te is approximately 120 pm, while those of Se and S are around 100 pm and 90 pm, respectively, leading to significant size differences of 20% and 30%. As a result, the calculated Fermi surface of NiTeS (Fig. S10c) reveals a more pronounced elongation of the ellipse along the k_y_ direction for the α band compared to the slightly distorted NiTeSe and relatively isotropic NiTe_2_, as shown in Fig. S10a,b. This observation suggests that the atomic size disparity disrupts the three-fold symmetry of NiTe_2_, resulting in a distinct in-plane anisotropic band structure in the NiTeSe and NiTeS alloys.


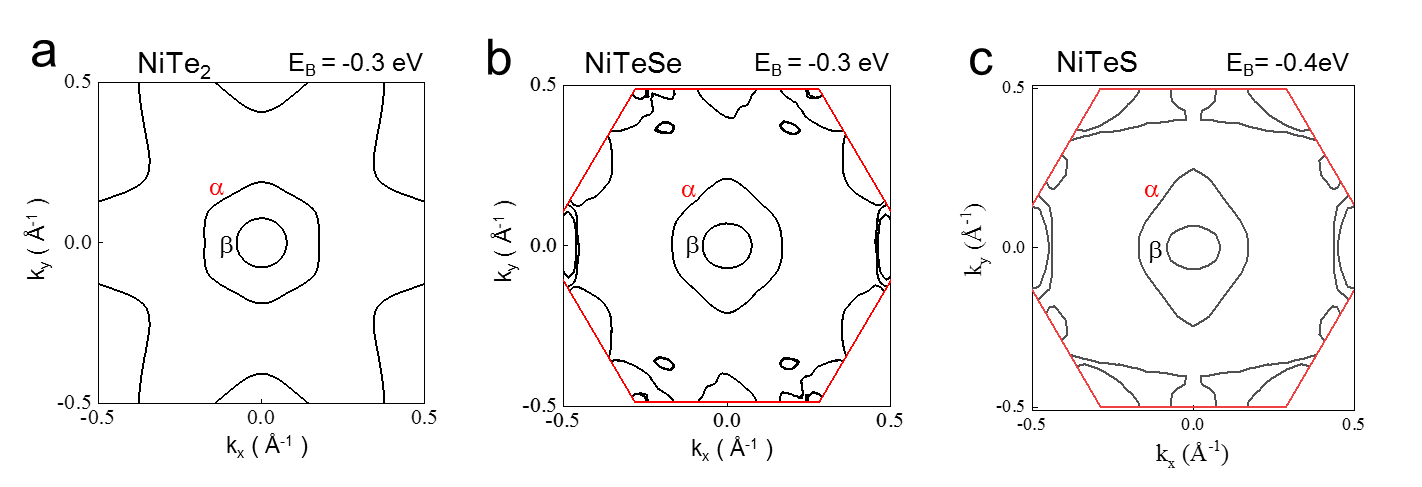


**Figure S10**. Calculated Fermi surface of a) NiTe_2_ and b) NiTeSe at *E*_B_ = -0.3 eV, and c) NiTeS at *E*_B_ = -0.4 eV.

Based on energy dispersive X-ray spectroscopy (EDS) measurement, the Te to Se ratio in NiTeSe was determined to be approximately 1 : 1, as shown in **Figure S11**.


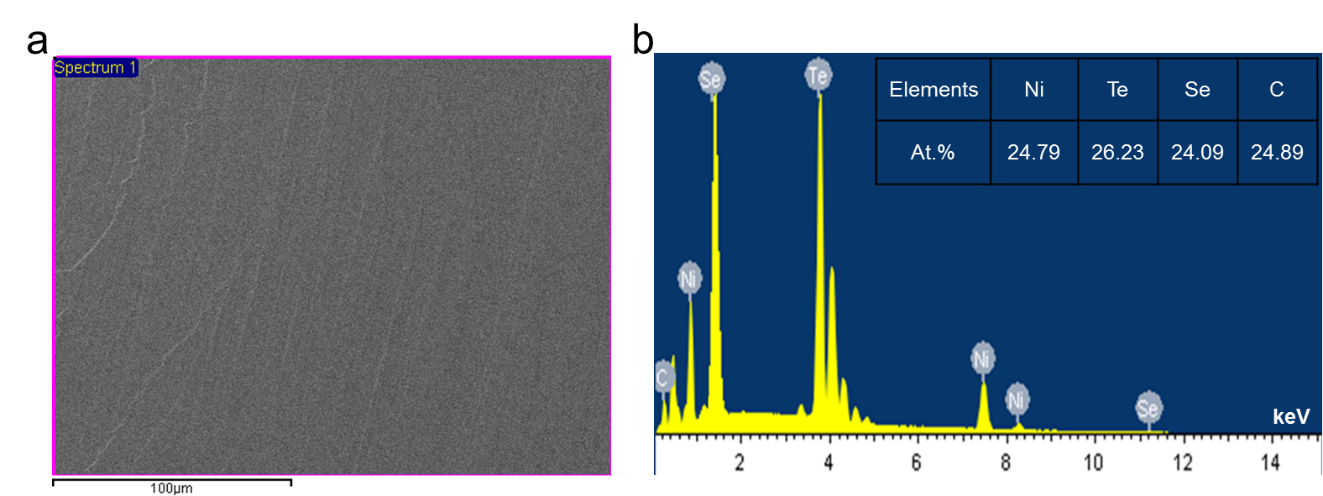


**Figure S11**. a) SEM image and b) EDS spectrum showing the typical composition of NiTeSe alloy.

References

[1] M. Bocquet, H. Lesnard, S. Monturet, N. Lorente, in *Computational Methods in Catalysis and Materials Science*, Wiley **2009**, pp. 199–219.

[2] J. Tersoff, D. R. Hamann, *Phys. Rev. Lett.* **1983**, *50*, 1998.
